# Preservation Methods Alter Carbon and Nitrogen Stable Isotope Values in Crickets (Orthoptera: Grylloidea)

**DOI:** 10.1371/journal.pone.0137650

**Published:** 2015-09-21

**Authors:** Fabiene Maria Jesus, Marcelo Ribeiro Pereira, Cassiano Sousa Rosa, Marcelo Zacharias Moreira, Carlos Frankl Sperber

**Affiliations:** 1 Programa de Pós-graduação em Ecologia, Departamento de Biologia Geral, Universidade Federal de Viçosa, Viçosa, MG, Brazil; 2 Universidade Federal do Espírito Santo, Centro de Ciências Agrárias, Departamento de Biologia, Alegre, ES, Brazil; 3 Universidade Federal do Triângulo Mineiro, Campus Iturama, Curso de Ciências Biológicas, Iturama, MG, Brazil; 4 Laboratório de Ecologia Isotópica, Centro de Energia Nuclear na Agricultura—CENA/USP, Piracicaba, SP, Brazil; Muséum national d’Histoire naturelle, FRANCE

## Abstract

Stable isotope analysis (SIA) is an important tool for investigation of animal dietary habits for determination of feeding niche. Ideally, fresh samples should be used for isotopic analysis, but logistics frequently demands preservation of organisms for analysis at a later time. The goal of this study was to establish the best methodology for preserving forest litter-dwelling crickets for later SIA analysis without altering results. We collected two cricket species, *Phoremia* sp. and *Mellopsis doucasae*, from which we prepared 70 samples per species, divided among seven treatments: (i) freshly processed (control); preserved in fuel ethanol for (ii) 15 and (iii) 60 days; preserved in commercial ethanol for (iv) 15 and (v) 60 days; fresh material frozen for (vi) 15 and (vii) 60 days. After oven drying, samples were analyzed for *δ* ^15^N, *δ* ^13^C values, N(%), C(%) and C/N atomic values using continuous flow isotope ratio mass spectrometry. All preservation methods tested, significantly impacted *δ* ^13^C and *δ* ^15^N and C/N atomic values. Chemical preservatives caused *δ* ^13^C enrichment as great as 1.5‰, and *δ* ^15^N enrichment as great as 0.9‰; the one exception was *M. doucasae* stored in ethanol for 15 days, which had *δ* ^15^N depletion up to 1.8‰. Freezing depleted *δ* ^13^C and *δ* ^15^N by up to 0.7 and 2.2‰, respectively. C/N atomic values decreased when stored in ethanol, and increased when frozen for 60 days for both cricket species. Our results indicate that all preservation methods tested in this study altered at least one of the tested isotope values when compared to fresh material (controls). We conclude that only freshly processed material provides adequate SIA results for litter-dwelling crickets.

## Introduction

The growing utilization of stable isotope analysis has helped set a rigorous empirical and theoretical basis for ecological studies of nutrient flow and trophic linkages [1–4]. The relationships among different stable carbon (^13^C/^12^C, expressed as *δ* ^13^C) and nitrogen (^15^N/^14^N, expressed as *δ* ^15^N) isotopes are widely used to investigate dietary habits of animals in order to determine their respective feeding niches [5–11]. Stable isotopes are naturally available in the environment and are ingested while feeding, thus an animal’s isotopic composition is indicative of its feeding habits throughout life [12, 13]. ^13^C/^12^C ratios can thus be used to identify consumer reliance on primary producers with different photosynthetic pathways, namely C3, C4, or CAM [14, 15]. A combination of ^13^C/^12^C and ^15^N/^14^N ratios is commonly used in animal studies to identify dietary composition, and to establish trophic position within both marine and terrestrial food webs [16–18]. Stable Isotope Analyses (SIA) has been used for studies of a broad scope of organisms, from unicellular phyto- and zooplankton [19], to seaweed [20, 21], higher plants [22], and several animal groups, including spiders [23], grasshoppers [7], termites [11], ants [2], flies [24], and vertebrates, including quail, sheep [25], and turtles [26], among others. To our knowledge, there has been no previous SIA studies in the context of cricket (Grylloidea) ecology.

Crickets are the most common Orthoptera in neotropical forest litter [27], which is the most productive and biodiverse *stratum* in these forests [28]. Knowledge of tropical forest invertebrates, especially neotropical crickets, is sparse. Neotropical cricket ecology is a recent field of study [29, 30], and to advance knowledge in this area a better understanding of cricket diet and feeding habits (i.e., beyond their general classification as ‘omnivorous’) [31] is needed. Although we know that crickets may feed on a range of items including plants, animals, and both living and dead organisms, we do not yet have data regarding feeding niche partitioning or variation. Stable isotope studies have strong potential as a tool for providing a more detailed picture of cricket feeding habits.

Stable isotopes may only be used as indicators of an organism’s diet if the isotope composition of analyzed samples corresponds exactly to that of the organism in the field, without aggregation of isotopes from any other source. Because sample processing for SIA relies on access to laboratory facilities, samples often cannot be analyzed in the field [20, 21, 25], and must instead be collected and stored for later analysis. If the chosen preservation technique alters isotopic values, then the SIA results may be improperly interpreted. A preferable alternative would be to avoid sample preservation altogether, by performing isotope analyses immediately after field collection [24], however, cricket collection is often done in remote regions, away from well-equipped labs. Rapid processing is thus unfeasible in the field, highlighting the need for specimen preservation techniques that avoid sample decomposition and subsequent alteration of SIA results.

Because litter crickets are easily startled and flee in response to substrate vibration [32], live (manual) capure is dificult. Passive sampling techniques are used to solve this limitation, commonly via pitfall traps filled with killing solution [30, 33]. This and other passive sampling methods are essential for studies at large spatial and temporal scales, or in studies that test local environmental drivers of biodiversity (i.e., factors that influence biodiversity, such as soil moisture, vegetation structure, resource availability), and should minimize researcher interference. Pitfall traps without killing solution do capture live crickets, but many can escape the trap. No-kill traps may also enable mesofaunal predators to feed on the intended study organisms (C.F. Sperber, pers. obs.).

Many types of killing solutions have been tested for efficiency in pitfall sampling, such as water and detergent [34], formaldehyde and ethylene glycol [33, 34], salt brines [35] and acetic acid [36]. However, ethanol solutions are considered as the most effective for cricket sampling due to rapid killing, which prevents escape [33, 37], and effective preservation of DNA [38, 39]. Ethanol solutions are also the most common preservation method in cricket taxonomic collections (e.g. Souza-Dias2015). For field sampling, Szinwelski et al. [39] recommended substitution of ethanol fuel as killiing agent in place of commercial ethanol, because the former is cheaper, logistically less cumbersome, and also preserves DNA.

Despite its effectiveness for preservation of whole cricket specimens and of cricket DNA, it remains questionable whether ethanol is an adequate preservation medium for cricket SIA. Ethanol is a lipophilic organic compound and thus could solubilize lipid compounds, altering the carbon isotope signature [2, 40]. Several studies have examined preservation effects on stable isotope ratios [19, 26, 41], with inconsistent results. For example, preservation in ethanol changes the isotope signature for some aquatic and marine organisms, including some species of fish, mollusk, seaweed, zooplankton, and anemone, but does not impact the isotope signatures of aquatic insects [19]. The unsuitability of ethanol has been challenged for vertebrates and invertebrates [19], as studies of ethanol-stored tissues of quail, sheep [25], turtles [26], insects [42] and macroinvertebrates [40] showed no changes in carbon isotope signature.

Samples subjected to organic solvents may have altered carbon isotopic signals due to loss of dissolved lipids and gain of solvent constituent carbon. By removing lipids, which are naturally highly depleted of ^13^C and rich in ^12^C) [43], ethanol may increase the ^13^C/^12^C sample values, thus amplifying the ^13^C signal. Carbon from ethanol preservatives might also be incorporated into the cricket bodies, altering the isotopic signals. Fuel ethanol is distinct from commercial ethanol because it is a complex mixture of flammable liquid and volatile hydrocarbons derived from petroleum, with carbonic chains varying from 4 to 12 carbon atoms as well as oxygenates and nitrogen compounds [44]; these additional components can alter *δ* ^15^N isotope signals. THus, *a priori* knowledge of how preservation methodologies interact with different sample types and the resulting impact on isotopic values is essential.

Viable alternatives to ethanol preservation may include freezing when immediate drying is not possible, but not all studies have considered the potential impacts of this method on stable isotope ratios. Among the studies that have tested effects of freezing, some found significant and even strong impacts on stable carbon and nitrogen isotope values [40, 45, 46], while others found none [21, 47].

Studies of the effects of different preservation methods (including freezing) on stable carbon and nitrogen values have produced highly variable results for both *δ* ^13^C and *δ* ^15^N values, ranging from no impact to over 2‰ difference between stored samples and controls [19, 45, 48]. Changes in carbon and nitrogen isotope values may be species-specific [20], and there may even be intra-specific differences [49] related to characteristics such as body size, cuticle thickness, or life stage-dependent changes in the proportion of fat reserves. Knowledge of how preservation methodologies interact with different sample types and the resulting impact on isotopic values is essential. Only then can we correctly interpret information regarding trophic ecology from isotope data based on preserved samples.

The aim of this study was to identify the best methodology for preservation of forest litter-dwelling crickets for later stable isotope evaluation. We compared the *δ* ^13^C and *δ* ^15^N isotope values of freshly dried crickets with those preserved via freezing or chemical storage, for either 15 or 60 days prior to analysis. If the preservation methods are appropriate for these organisms, then SIA results should not differ between freshly dried and preserved cricket samples.

We expected that samples preserved in ethanol (both fuel and commercial) would vary in isotopic carbon and nitrogen values compared to controls. Further, we anticipated that the distance of carbon and nitrogen isotope values in ethanol, compared to control samples, should increase with storage time. Finally, we expected that frozen samples, which are not subjected to chemical compounds, should not differ from freshly processed material.

## Materials and Methods

### Collection of crickets

We collected two species of forest litter cricket (Orthoptera: Grylloidea), *Phoremia* sp. (Trigonidiidae: Nemobiinae) (S1A Fig) and *Mellopsis doucasae* Mews & Sperber, 2010 (Phalangopsidae: Luzarinae) (S1B Fig). These species are not endangered or protected. They occur in Atlantic forest remnants in the region of Viçosa, Minas Gerais state, southeastern Brazil, where the dominant vegetation type is secondary (regrowth) seasonal semi-deciduous montane forest [50]. Forest litter crickets have been observed feeding on fruits, arthropods and leaves (C.F. Sperber, pers. obs.). We chose these species because although they share the same microhabitat, *Phoremia* sp. is diurnal and *M. doucasae* is nocturnal (M.R. Pereira, pers. obs.). They are also phylogenetically distant [51, 52], thus we expected that they should present different isotopic signals.

Collections were carried out from the 4 th to 19 th July 2013. Crickets were collected manually (live capture) on leaf litter using wide-mouth (15 cm diameter) jars. Collections were done daily for about four hours per day, until we gathered approximately 1000 individuals. All collections were done in a 60 ha semideciduous Atlantic forest remnant, called “Mata da Biologia”, on the Campus of the Federal University of Viçosa, Viçosa, MG (S 20°45’30”—W 42°51’50”). No specific permissions were required to collect insects in the area at the time of the study. Our group has general authorization to collect insects in the entire Brazilian territory via a Permanent License for Collection of Zoological Material, provided by the Brazilian Institute of the Environment and Natural Resources (Instituto Brasileiro do Meio Ambiente e dos Recursos Naturais Renováveis—IBAMA) for CFS, number 553948. Species determination was done with the aid of a specialist (Marcelo R. Pereira); voucher specimens were deposited in the Orthopteran collection of the Orthoptera Laboratory in the General Biology Department, (Coleção de Orthoptera do Laboratório de Orthoptera, Departamento de Biologia Geral), affiliated with the Regional Museum of Entomology at the Federal University of Viçosa (Museu Regional de Entomologia, Universidade Federal de Viçosa—UFVB), Viçosa, Minas Gerais, Brazil. Crickets were brought to the laboratory, provided with water on a dampened piece of cotton, and kept alive and unfed for two days in order to empty the digestive tract.

### Preservation and processing

After two days of food deprivation, crickets were killed by exposure to −20°C for up to 20 min. As soon as the crickets stopped moving, they were taken out of the refrigerator, so as to prevent freezing them. This killing method aimed to prevent suffering while avoiding freezing of the samples. After killing, each cricket was rinsed with distilled water, to wash dirt and particles attached to the cricket’s body surface, and stored in glass vials in pooled samples of eight *Phoremia* sp. adults or four *M. doucasae* immatures, totaling 70 samples for each species. Life stages were selected as available in the field. These samples were divided equally among seven treatments for a total of 10 sample replicates per treatment, as follows: (i) freshly processed (controls); (ii) preserved in fuel ethanol for 15 days; (iii)preserved in fuel ethanol for 60 days; (iv) preserved in 92.8% (96° GL) commercial ethanol for 15 days; (v) preserved in 92.8% (96° GL) commercial ethanol for 60 days; (vi) fresh material frozen for 15 days; (vii) fresh material frozen for 60 days. All glass vials used in the ethanol treatments were properly closed and stored at room temperature, and protected from light and heat. Immediately after killing (controls) or after preservation period, the samples were oven-dried in open glass vials at 60°C for 72 hours. After drying, each pooled sample was ground and sieved (100 *μ*m mesh) separately, prior to isotope analysis, so as to achieve complete homogenization of each sample. Therefore, individual insects of each sample were ground together and analyzed as a homogenized batch. After finishing homogenization of each sample, all laboratory tools where detergent-washed, rinsed and dried, before processing the next sample.

### Stable isotope analysis

We performed continuous flow isotope ratio mass spectrometry (CF-IRMS) for sample analysis using a Delta Plus mass spectrometer (Thermo Scientific) coupled to a Carlo Erba CHN 1110 elemental analyzer (Thermo Scientific). Analyses were performed at the Isotope Ecology Laboratory at the Center for Nuclear Energy in Agriculture, CENA, University of São Paulo (Laboratório de Ecologia Isotópica do Centro de Energia Nuclear na Agricultura—CENA, University of São Paulo, São Paulo), São Paulo state, Brazil. Briefly, organic matter (i.e., milled cricket samples) was converted into gas by fully dry combustion, generating N_2_ and CO_2_, which were then purified in the elemental analyzer through a chromatographic separation column in ultrapure helium carrier, and sequentially admitted to the mass spectrometer by means of an interface (Conflo II, Thermo Scientific). The ^15^N/^14^N and ^13^C/^12^C isotope ratios were evaluated after separation of molecules according to isotope mass, and finally compared to the calibrated gas ratios using Vienna PeeDee Belemnite (VPDB) limestone and atmospheric nitrogen as international standards for *δ* ^13^C and *δ* ^15^N, respectively. These working standards were calibrated using NBS-19 and NBS-22, and AIEA-N1 and IAEA-N2 as reference materials and the estimated analytical precision of these measurements was 0.1‰ for carbon and 0.2‰ for nitrogen based on the standard deviation of WS replicates during the runs. Results are expressed in “delta” notation (*δ* ^13^C, *δ* ^15^N) in parts per thousand (‰) as relative deviations of the isotope ratios compared to standards, using the following formula:

$$\begin{array}{c} \delta X=(\frac{R_{sample}}{R_{standard}}-1)*1000 \end{array}$$ (1)

where *R*_*sample*_ and *R*_*standard*_ are the ^13^C/^12^C and ^15^N/^14^N ratios for the sample and international standards, respectively. X represents the “heavy” isotopes ^13^C or ^15^N. The *δ*X values denote isotopic enrichment or depletion relative to the standard; a more positive “*δ*” value is isotopically enriched, meaning that the sample contains proportionally more of the “heavy” stable isotope (^13^C or ^15^N). The weight percentages of carbon and nitrogen output from the the instrument mentioned above were converted to C/N atomic values.

### Statistical analyses

We used analyses of variance (ANOVA) with preservation method as an explanatory factor. We ran an independent analysis for each response variable (*δ* ^13^C, *δ* ^15^N, %C, %N and C/N) by adjusting generalized linear models (GLM). Modeling was performed using R [53], with normal error distribution confirmed by residual analysis.

We evaluated which factor levels (preservation methods) were significantly different through contrast analysis, by aggregating levels and comparing change in deviance [54]. If the aggregated level did not significantly alter the deviance explained by the model, the levels were pooled together (amalgamated), simplifying the model. We repeated this procedure until attaining a minimum adequate model for each response variable (*δ* ^13^C, *δ* ^15^N, %C, %N and C/N) by stepwise omission of non-significant terms.

Adequate preservation methods should result in stable isotope signatures that do not differ from freshly processed material. We interpreted any differences between preserved and freshly processed material as an indication of altered isotope signature due to inadequate preservation methods. We evaluated storage time effects on SIA values by comparing samples preserved in the same solution types, independent of control results.

## Results

**Fig 1:**
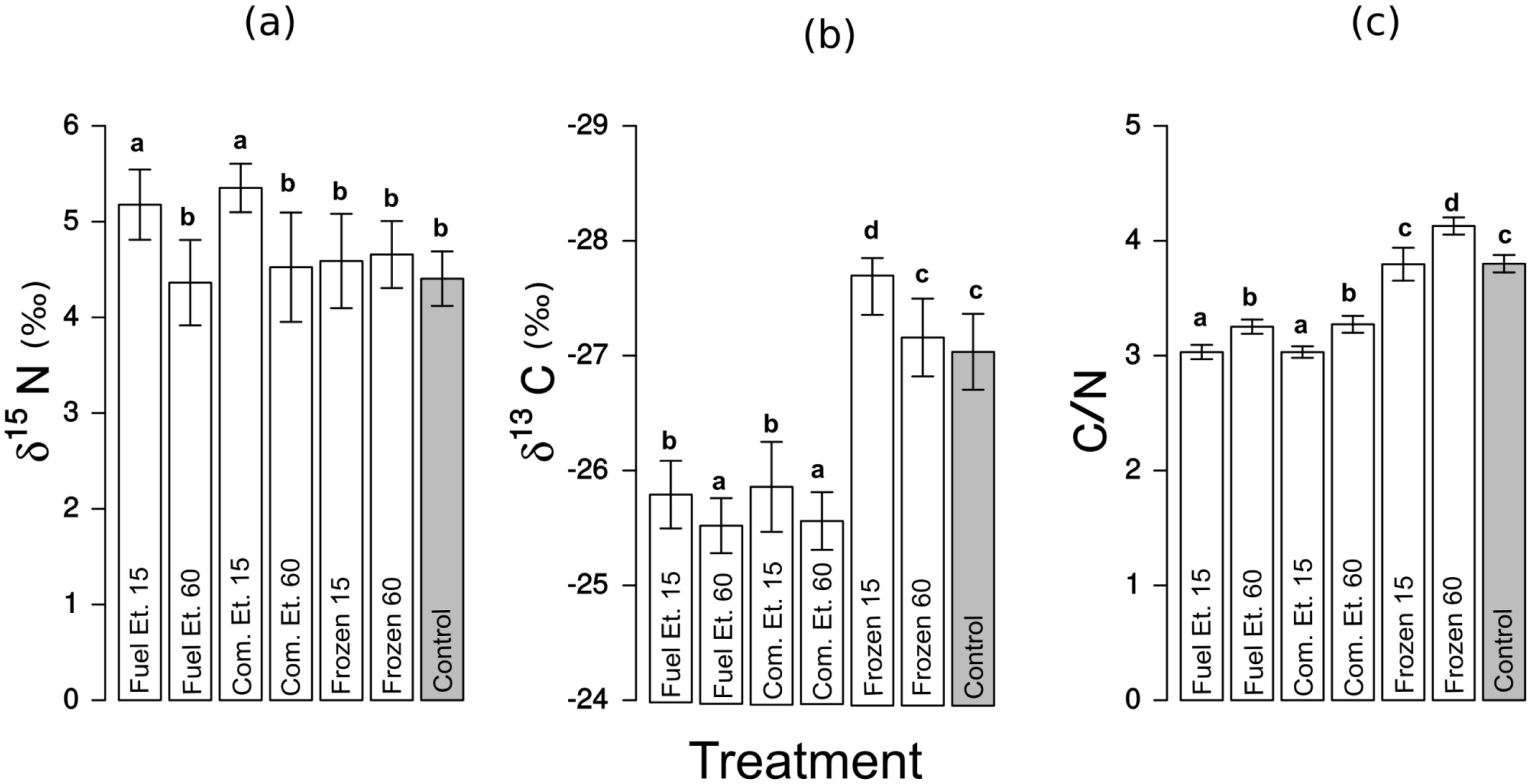
Carbon and nitrogen stable isotope values, and C/N atomic values for *Phoremia* sp. Mean ± standard deviation of (a) *δ* ^15^N, (b) *δ* ^13^C, and (c) C/N atomic values from the following treatments: Fuel Et. 15—preserved in fuel ethanol for 15 days; Fuel Et. 60—preserved in fuel ethanol for 60 days; Com. Et. 15—preserved in 92.8% commercial ethanol for 15 days; Com. Et. 60—preserved in 92.8% commercial ethanol for 60 days; Frozen 15—frozen for 15 days; Frozen 60—frozen for 60 days; Control—freshly processed material (highlighted in gray). Different letters indicate significant differences between treatment groups (P < 0.05).

**Fig 2:**
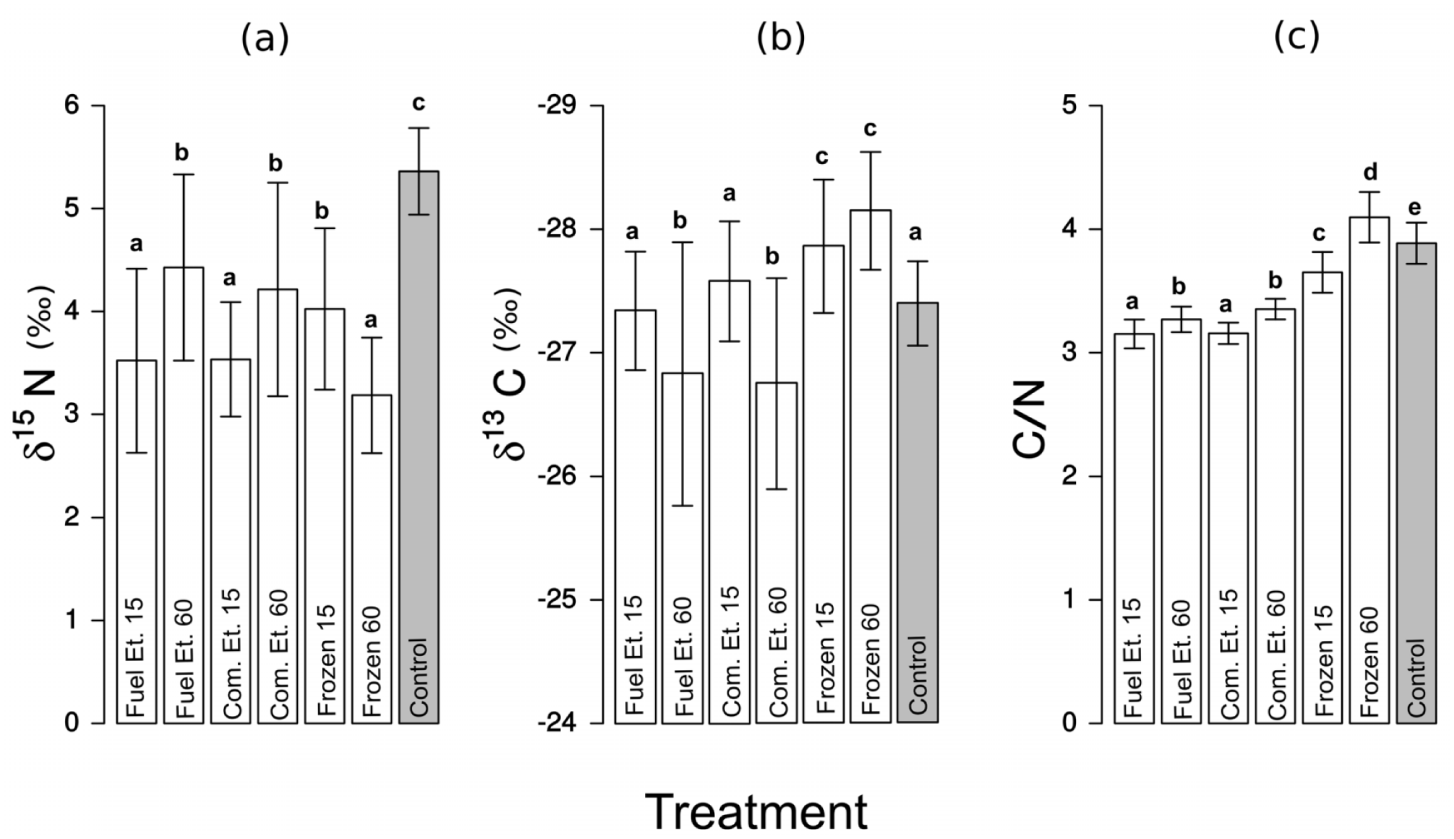
Carbon and nitrogen stable isotope values, and C/N atomic values for *Mellopsis doucasae*. Mean ± standard deviation of (a) *δ* ^15^N, (b) *δ* ^13^C, and (c) C/N atomic values from the following treatments: Fuel Et. 15—preserved in fuel ethanol for 15 days; Fuel Et. 60—preserved in fuel ethanol for 60 days; Com.Et. 15—preserved in 92.8% commercial ethanol for 15 days; Com.Et. 60—preserved in 92.8% commercial ethanol for 60 days; Frozen 15—frozen for 15 days; Frozen 60—frozen for 60 days; Control—freshly processed material (highlighted in gray). Different letters indicate significant differences between treatment groups (P < 0.05). All preservation methods resulted in significant ^15^N depletion compared to controls.

Evaluation of external morphology of both frozen samples and those preserved in ethanol (fuel and commercial) revealed no signs of decomposition by the end of the experiment (60 days). However, most preservation methods altered *δ* ^15^N, *δ* ^13^C and C/N values in both species compared to controls (Figs 1 and 2, Table 1, sequences of statistical model simplifications for contrast analyses are presented in S1 Table and S2 Table). Although the effects of preservation method on isotope values differed between species (Figs 1 and 2), total content of C, N, and C/N atomic values were similar (Table 1).

**Table 1:** Carbon and nitrogen stable isotope values, N(%), C(%) and C/N atomic values. Mean ± SD for *δ* ^15^N, *δ* ^13^C isotopic values, nitrogen and carbon total content (%), and C/N atomic values of cricket samples subjected to different preservation methods.

Species	Methods	Mean ± SD (n = 10)				
		*δ* ^15^N‰	*δ* ^13^C‰	N (%)	C (%)	C/N
*Phoremia* sp.	Control—Freshly processed	4.4 ± 0.3	-27.0 ± 0.3	11.7 ± 0.2	44.4 ± 0.6	4.4 ± 0.1
	Freezer—15 days	4.6 ± 0.5	-27.6 ± 0.2	11.9 ± 0.4	45.1 ± 0.7	4.4 ± 0.1
	Freezer—60 days	4.7 ± 0.3	-27.2 ± 0.3	11.2 ± 0.2	46.3 ± 0.4	4.8 ± 0.1
	Ethanol fuel—15 days	5.2 ± 0.4	-25.8 ± 0.3	13.8 ± 0.3	41.8 ± 0.6	3.5 ± 0.1
	Ethanol fuel—60 days	4.4 ± 0.4	-25.5 ± 0.2	13.2 ± 0.5	43.1 ± 1.3	3.8 ± 0.1
	Ethanol (92,8%)—15 days	5.3 ± 0.2	-25.9 ± 0.4	13.9 ± 0.3	42.1 ± 0.4	3.6 ± 0.1
	Ethanol (92,8%)—60 days	4.5 ± 0.6	-25.6 ± 0.2	13.4 ± 0.3	43.8 ± 0.5	3.8 ± 0.1
*Mellopsis doucasae*	Control—Freshly processed	5.4 ± 0.4	-27.4 ± 0.3	11.8 ± 0.3	45.6 ± 0.9	4.5 ± 0.2
	Freezer—15 days	4.0 ± 0.8	-27.9 ± 0.5	12.1 ± 0.5	44.2 ± 0.8	4.3 ± 0.2
	Freezer—60 days	3.2 ± 0.6	-28.1 ± 0.5	11.3 ± 0.5	46.1 ± 0.7	4.8 ± 0.2
	Ethanol fuel—15 days	3.5 ± 0.9	-27.3 ± 0.5	13.7 ± 0.5	43.2 ± 0.3	3.7 ± 0.1
	Ethanol fuel—60 days	4.4 ± 0.9	-26.8 ± 1.0	13.3 ± 0.5	43.3 ± 0.5	3.8 ± 0.1
	Ethanol (92,8%)—15 days	3.5 ± 0.6	-27.6 ± 0.5	13.5 ± 0.4	42.7 ± 0.6	3.7 ± 0.1
	Ethanol (92,8%)—60 days	4.2 ± 1.0	-26.7 ± 0.9	13.0 ± 0.2	43.5 ± 0.7	3.9 ± 0.1

### *δ* ^15^N values

The *δ* ^15^N values of freshly processed samples (controls) for *Phoremia* sp. did not differ from samples submitted to four of the preservation methods (both frozen samples (i.e., 15 and 60 days) and samples preserved for 60 days in fuel or commercial ethanol) (P > 0.07; S1 Table), however values did differ from samples preserved for 15 days in both commercial and fuel ethanol (F_1,68_ = 40.35, P < 0.0001, Fig 1a). For samples preserved in ethanol for 15 days, there was mean enrichment of *δ* ^15^N up to 0.9‰ compared to all other preservation methods and controls (Fig 1a; Table 1). For *M. doucasae*, *δ* ^15^N values from all preservation methods were significantly depleted compared to controls (F_2,67_ = 26.23, P < 0.0001, Fig 2a; results of contrast analyses in S2 Table), with mean depletion ≤ 2.2‰ (Fig 2a; Table 1).

### *δ* ^13^C values

*δ* ^13^C values of preserved *Phoremia* samples differed significantly from controls (F_3,66_ = 168.8, P < 0.0001), with the exception of samples frozen for 60 days (F_1,65_ = 0.87, P = 0.35, Fig 1b; S1 Table). Mean *δ* ^13^C enrichment of up to 1.5‰ occurred in ethanol-preserved samples, while frozen samples showed depletion of up to 0.6‰ compared to controls (Fig 1b; Table 1).

For *M. doucasae*, *δ* ^13^C samples preserved in commercial and fuel ethanol solutions for 15 days did not differ from controls (F_1,65_ = 0.71, P = 0.40), but did differ from all other preservation methods (F_2,67_ = 18.06, P < 0.0001, Fig 2b; S2 Table). Samples preserved in ethanol for 60 days had mean *δ* ^13^C enrichment of up to 0.6‰ compared to controls; for frozen samples, there was a mean *δ* ^13^C depletion of up to 0.7‰ (Fig 2b; Table 1).

### C/N ratio

For *Phoremia* sp., C/N atomic values from samples frozen for 15 days did not differ from controls (F_1,64_ = 0.01, P = 0.91), however all other preservation methods did differ from controls (F_3,66_ = 572.75, P < 0.001) (Fig 1c; Table 1; S1 Table). All preservation methods for *M. doucasae* resulted in C/N atomic values that significantly differed from controls (F_5,64_ = 88.2, P < 0.001, Fig 1c; Table 1; S2 Table).

### Storage time

Storage time prior to processing had a significant effect on isotope values and C/N atomic values for both cricket species, with a few exceptions (Figs 1a, 1b, 1c, and 2a, 2b, 2c; Table 1). For all but one preservation method, *Phoremia* sp. showed no significant differences in C or N isotope values between different chemical solutions (fuel or commercial ethanol) when storage time was equal (*δ* ^15^N: Com.Et.15 = Fuel Et.15, F_1,66_ = 0.95, P = 0.33; *δ* ^13^C: Com.Et.15 = Fuel Et.15, F_1,64_ = 0.25, P = 0.61; *δ* ^13^C: Com.Et.60 = Fuel Et.60, F_1,63_ = 0.09, P = 0.77; S1 Table). The exception was *δ* ^15^N in samples stored for 60 days, in which fuel-ethanol preserved sampled yielded different values than did commercial ethanol-stored samples. Fuel ethanol stored samples (60 d) did not differ from frozen or control samples (P > 0.82; S1 Table). There was 0.8‰ depletion of *δ* ^15^N in samples preserved in ethanol for 60 days compared to 15 days. *δ* ^13^C values were increased by 0.3‰ in samples stored in commercial and fuel ethanol for 60 days, compared to those stored for 15 days (Fig 1a and 1b; Table 1).

For *M. doucasae*, there were no differences in *δ* ^15^N values between preservation solutions (fuel or commercial ethanol) when storage time was equal (*δ* ^15^N: Com.Et.15 = Fuel Et.15, F_1,64_ = 0.01, P = 0.97; Com.Et.60 = Fuel Et.60, F_1,63_ = 0.39, P = 0.54). There were no differences in *δ* ^13^C values between chemical solutions (fuel vs. commercial ethanol) and controls when storage time was equal (*δ* ^13^C: Com.Et.15 = Fuel Et.15 = Control, F_1,65_ = 0.71, P = 0.40; Com.Et.60 = Fuel Et.60, F_1,64_ = 0.07, P = 0.79; S2 Table). Longer storage time in ethanol led to enrichment in *δ* ^15^N (0.9‰ and 0.7‰, respectively for commercial and fuel ethanol) and *δ* ^13^C (0.5‰ and 0.9‰, respectively for commercial and fuel ethanol) (Fig 2a and 2b; Table 1).

There were no differences in C/N atomic values between ethanol preservation solutions in either cricket species when storage time was equal (*Phoremia* sp.– C/N: Com.Et.15 = Fuel Et.15, F_1,63_ = 0.0, P = 1; Com.Et.60 = Fuel Et.60, F_1,65_ = 0.33, P = 0.56, Fig 1c; S1 Table; *M. doucasae*—C/N: Com.Et.15 = Fuel Et.15, F_1,63_ = 0.09, P = 0.93; Com.Et.60 = Fuel Et.60, F_1,65_ = 1.69, P = 0.2, Fig 2c; S2 Table).

## Discussion

This study presented the first evidence of preservation method-dependent shifts in *δ* ^13^C and *δ* ^15^N in cricket specimens, and contributed with novel and useful information on the use of preserved samples in SIA studies. Of particular relevance was the notion that even methods sometimes used for control groups, i.e. freezing [20, 24, 55, 56], may alter SIA results.

Our results reinforced previous studies showing that storage method can have very different impacts on stable isotope values depending on preservative type and sample taxon [20, 49, 57]. For example, though some studies have proposed efficient preservation methods for SIA (e.g., for Isoptera: EtOH 80% and NaCl [58]; for Diptera and Hymenoptera: EtOH ≥ 70% for *δ* ^15^N values [2, 24]; for aquatic insects: EtOH 80% [42] and EtOH 75% [19]), our results agreed with a large proportion of alternative studies demonstrating that such preservatives may alter either *δ* ^15^N or *δ* ^13^C values, or both [19–21, 45, 47–49, 59–61].

The two preservation methods tested in this study similarly affected *δ* ^13^C values and C/N atomic values, with chemical preservatives generally producing depletion and freezing producing enrichment (after 60 days) in both cricket species. *δ* ^13^C was enriched over time in both cricket species, with *M. doucasae* (1.5‰) becoming more enriched than *Phoremia* sp. (0.6‰). The range of enrichment values (0.6-1.5‰), irrespective of preservation time, was similar to the ranges found for ants [2], fish, octopus and kelp [21], fish muscle and liver [47], zooplankton [45], and bird tissues and blood (0.7-1.5‰) [61]. *δ* ^15^N values also varied between species, with depletion of up to 2.2‰ in *M. doucasae* and enrichment of 0.9‰ in *Phoremia* sp. preserved in ethanol for 15 days. Different mechanisms are proposed to explain how preservation techniques may affect ^13^C/^12^C and ^15^N/^14^N ratios in samples. One hypothesis attributes enrichment in both to assimilation of heavier isotopes present in the preserving agents [19, 25]. Both preservatives (fuel ethanol and commercial ethanol) are carbon-based chemicals with characteristic *δ* ^13^C signatures, and once preserved samples are immersed, their signature may shift toward that of the preservative. Another hypothesis suggests the loss of molecules carrying the “lighter” isotope (e.g., lipid molecules or nitrogenous excreta) [5, 25]. By removing lipids, which are naturally highly depleted of ^13^C (and rich in ^12^C) [43], ethanol may increase the ^13^C/^12^C ratio in sample, thus amplifying its ^13^C signal. According to Post et al. [62], the C/N ratio is a strong predictor of lipid content in animals. Thus, if the lipid content or C/N ratio is high (C/N > 4 for terrestrial animals [62]), then lipid concentrations are also sufficiently high to assume that extraction will affect *δ* ^13^C values [63–65]. The C/N atomic values in this study were greater than four in all treatments that were not preserved in ethanol, indicating high lipid content in the crickets, and reinforcing the hypothesis of lipid leaching in ethanol-preserved samples.

We noted a striking increase in *δ* ^13^C in all ethanol samples, which were greater in *Phoremia* sp. (Fig 1(b)—up to 1.5‰) than in *M. doucasae* (Fig 2(b)—up to 0.7‰). We attribute the changes in these values to the leaching of lipid in the smaller body-sized *Phoremia* sp. preserved in ethanol solution. Smaller bodies result in larger surface area to volume ratios, leading to stronger solvent action [66–68].

Freezing, a common preservation method, altered isotopic values in both cricket species. Freezing caused an increase in C/N atomic values (Figs 1c and 2c), and a decrease in both *δ* ^13^C (Figs 1b and 2b) and *δ* ^15^N (Fig 2a). Although some previous studies found no effects of freezing on SIA results [20, 21, 43, 47], others detected shifts in the *δ* ^15^N or *δ* ^13^C values, or both [3, 45, 46, 48, 57]. Feuchtmayr and Grey [45] and Dannheim et al. [46] suggested that SIA shifts in frozen samples may result from the breakdown of cells and subsequent loss of cytosol, as well as metabolic degradation by free enzymes and microorganisms. This process results in carbon and nitrogen leaching when thawed, but there is no direct evidence of mechanical cell destruction and cytosol loss in normally frozen samples (- 20°C) is causing the observed decrease in *δ* ^13^C and *δ* ^15^N [45, 46]. That cytosol components are indeed isotopically lighter is only speculation. Our results show that storage time prior to processing may enhance the effects of chemical preservatives on C and N isotopes. Longer storage time in ethanol (both commercial and fuel) produced slight enrichment of *δ* ^13^C in both cricket species, with species-dependent effects on *δ* ^15^N.

Lecea et al. [59] also observed that long-term effects of the preservative solutions were species-dependent. They compared *δ* ^13^C values between two zooplanktonic species stored in EtOH for 1, 3 or 9 months and found an enrichment peak at 3 months, with subsequence depletion occurring after 9 months. Our storage periods were shorter, yet still sufficient to provoke similar shifts in SIA profile. For *M. doucasae*, a storage period of 15 days caused a decrease of up to 1.8‰ in *δ* ^15^N values compared to controls. However, when the storage time was 60 days *δ* ^15^N values increased, and the difference compared to controls fell to 1.1‰.

The similarities in total C and N content and C/N atomic values indicates similar chemical composition between species. All preservation methods for *Phoremia* sp. changed the C/N atomic values compared to controls, with the exception of freezing for 15 days (Figs 1c and 2c). Contrasts revealed a subtle difference among treatment levels in C/N atomic values, but mean value profiles were similar among cricket species. Furthermore, the SIA shifts caused by the different preservation methods were similar between species. We interpret these similarities as evidence for consistent SIA shift patterns due to preservation methods, indicating that the differences in effects of preservation methods between species were not experimental artifacts. They instead result from effectively different chemical processes, brought about by the interaction between preservation method and intrinsic characteristics of each species.

Some authors suggest the use of a correction factor for preserved samples to account for eventual SIA shifts, especially when differences in isotopic values between preserved and unpreserved samples are minor compared to the assumed enrichment between trophic levels [16, 26, 56]. However, we agree with other authors [20, 40, 47, 64, 69] that advise against correction factor extrapolation beyond the studied species SIA shifts caused by cricket preservation would confound critical ecological information, which suggests avoidance of sample preservation altogether.

## Conclusions

This study showed that preservation methods such as freezing and ethanol storage significantly affect stable isotope values in cricket samples. Our results also revealed SIA value shifts along storage time, and suggest interspecific differences in the effects of storage time on SIA results. Further, we detected an idiosyncratic interaction of species identity with storage method on SIA results. We therefore recommend that storage of cricket samples for SIA should be avoided, and that samples stored for different lengths of time may not be directly comparable. Only freshly dried samples should be used for production of reliable SIA results.

## Supporting Information

S1 Fig Picture of the studied cricket species. A: Male *Phoremia* sp. (Orthoptera: Trigonidiidae: Nemobiinae); B: male *Mellopsis doucasae* (Orthoptera: Phalangopsidae: Luzarinae).
(PDF)

S1 Table Models and contrasts used to inspect the effects of preservation methods on the *δ* ^15^N, *δ* ^13^C isotopic values, %N, %C total content and C/N atomic values of *Phoremia* sp. samples. Model simplification was performed by backward term extraction, removing one term at a time, performing consecutive one degree of freedom contrast analyses. Minimum Adequate Model’s significance was evaluated contrasting it with the null model.
(PDF)

S2 Table Models and contrasts used to inspect the effects of preservation methods on the *δ* ^15^N, *δ* ^13^C isotopic values, %N, %C total content and C/N atomic values of *Mellopsis doucasae* sp. samples. Model simplification was performed by backward term extraction, removing one term at a time, performing consecutive one degree of freedom contrast analyses. Minimum Adequate Model’s significance was evaluated contrasting it with the null model.
(PDF)

S1 Dataset All data included in Results and S1–S2 Tables. (CSV)
