# Supplementary material for: Preservation Methods Alter Carbon and Nitrogen Stable Isotope Values in Crickets (Orthoptera: Grylloidea)
Source: PLoS One. 2015 Sep 21;10(9):e0137650. doi: 10.1371/journal.pone.0137650 (PMC4577105; doi:10.1371/journal.pone.0137650)

S1 Fig. Picture of the studied cricket species. A: Male *Phoremia* sp. (Orthoptera: Trigonidiidae: Nemobiinae); B: male *Mellopsis doucasae* Mews & Sperber, 2010 (Orthoptera: Phalangopsidae: Luzarinae) collected in Atlantic forest remnants in the region of Viçosa, Minas Gerais state, southeastern Brazil.

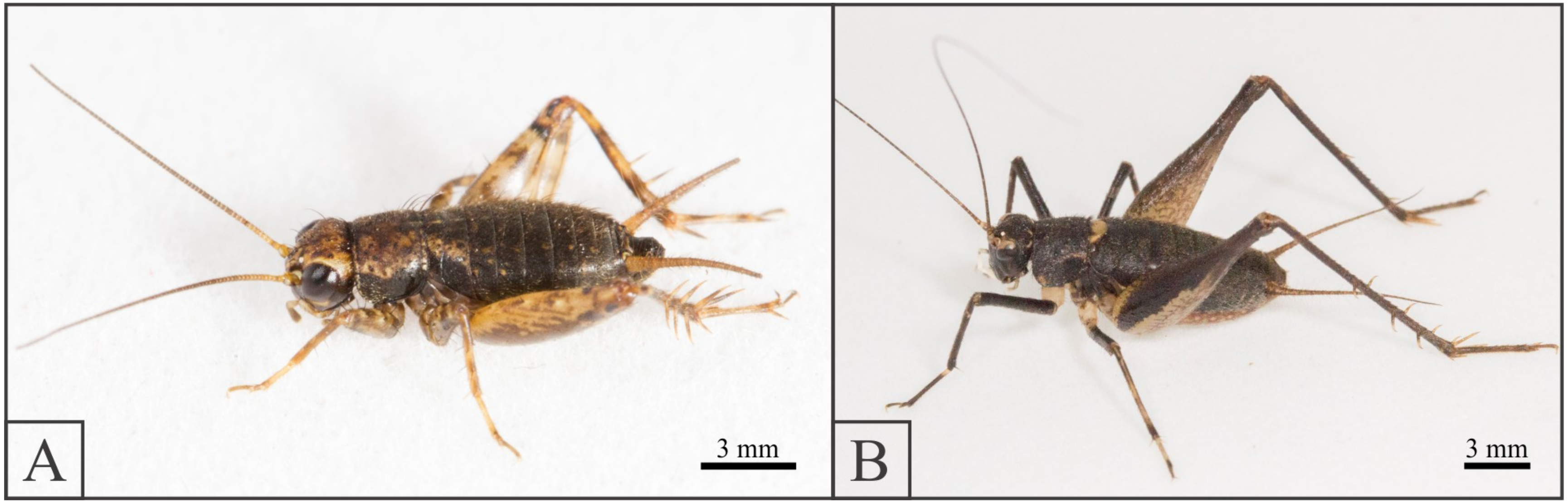

Supplement: S1 Fig — A: Male Phoremia sp. (Orthoptera: Trigonidiidae: Nemobiinae); B: male Mellopsis doucasae (Orthoptera: Phalangopsidae: Luzarinae). (PDF) [file pone.0137650.s001.pdf]
