# Supplementary material for: Preservation Methods Alter Carbon and Nitrogen Stable Isotope Values in Crickets (Orthoptera: Grylloidea)
Source: PLoS One. 2015 Sep 21;10(9):e0137650. doi: 10.1371/journal.pone.0137650 (PMC4577105; doi:10.1371/journal.pone.0137650)
Supplement: S1 Table — Model simplification was performed by backward term extraction, removing one term at a time, performing consecutive one degree of freedom contrast analyses. Minimum Adequate Model’s significance was evaluated contrasting it with the null model. (PDF) [file pone.0137650.s002.pdf]

S1 Table. Models and contrasts used to inspect the effects of preservation method on the $\delta^{13}C$, $\delta^{15}N$ isotopic values, C(%) and N(%) total content and C/N ratio of *Phoremia* sp. samples. Treatment levels were: Fuel Et.15 – preserved in fuel ethanol for 15 days; Fuel Et.60 – preserved in fuel ethanol for 60 days; Com. Et.15 – preserved in 92.8% commercial ethanol for 15 days; Com. Et.60 – preserved in 92.8% commercial ethanol for 60 days; Frozen 15 – frozen for 15 days; Frozen 60 – frozen for 60 days; Control – freshly processed material. Model simplification was performed by backward term extraction, removing one term at a time, performing consecutive one degree of freedom contrast analyses. Terms returned to the model if their removal provoked a change of deviance with $p<0.05$. Factor level contrasts were performed testing the amalgamation of treatment levels with the most similar mean values (=a posteriori amalgamation). When contrast was not significant, we repeated the procedure of term deletion of factor level amalgamation, up to achieving the Minimum Adequate Model (MAM), where any further amalgamation would lead to significant change in deviance. MAM's significance was evaluated contrasting it with the null model (nm). Amalgamation is indicated by the symbol +. All models with normal (Gaussian) error distribution. NS= $p > 0.05$; *= $p < 0.01$; **= $p < 0.001$; ***= $p < 0.0001$.

$\delta^{15}N$	$\delta^{15}N$				
**Models**	**Contrasts**	**df**	**F**	***p***	
m1: y ~ treatments (Fuel Et.15, Fuel Et.60, Com. Et.15, Com. Et.60, Frozen15, Frozen60, Control)	m2 x m1	1	0.06	0.81	NS
m2: y ~ treatments (Fuel Et.15, Fuel Et.60+Control, Com. Et.15, Com. Et.60, Frozen15, Frozen60)	m3 x m2	1	0.13	0.72	NS
m3: y ~ treatments (Fuel Et.15, Fuel Et.60+Control, Com. Et.15, Com. Et.60+Frozen 15, Frozen 60)	m4 x m3	1	0.42	0.52	NS
m4: y ~ treatments (Fuel Et.15, Fuel Et.60+Control, Com. Et.15, Com. Et.60+Frozen15+Frozen60)	m5 x m4	1	0.97	0.33	NS
m5: y ~ treatments (Fuel Et.15+Com. Et.15, Fuel Et.60+Control, Com. Et.60+Frozen15+Frozen60)	m6 x m5	1	3.21	0.08	NS
m6: y ~ treatments (Fuel Et.15+Com. Et.15, Fuel Et.60+Control+Com. Et.60+Frozen15+Frozen60)	nm x m6 (MAM)	1	27.08	< 0.0001	***
nm: y ~ 1	Error	68			

Continues

Table S1. (Cont.)

$\delta^{13}C$

**Models**
m1: y ~ treatments (Fuel Et.15, Fuel Et.60, Com. Et.15, Com. Et.60, Frozen15, Frozen60, Control)
m2: y ~ treatments (Fuel Et.15, Fuel Et.60+Com. Et.60, Com. Et.15, Frozen15, Frozen60, Control)
m3: y ~ treatments (Fuel Et.15+Com. Et.15, Fuel Et.60+Com. Et.60, Frozen15, Frozen60, Control)
m4: y ~ treatments (Fuel Et.15+Com. Et.15, Fuel Et.60+Com. Et.60, Frozen15, Frozen60+Control)
nm: y ~ 1

$\delta^{13}C$

**Contrasts**	**df**	**F**	***p***	
m2 x m1	1	0.09	0.77	NS
m3 x m2	1	0.26	0.61	NS
m4 x m3	1	0.86	0.36	NS
nm x m4 (MAM)	3	168.67	<0.0001	*
Error	66			

N(%)

**Models**
m1: y ~ treatments (Fuel Et.15, Fuel Et.60, Com. Et.15, Com. Et.60, Frozen15, Frozen60, Control)
m2: y ~ treatments (Fuel Et.15+Com. Et.15, Fuel Et.60,Com. Et.60, Frozen15, Frozen60, Control)
m3: y ~ treatments (Fuel Et.15+Com.Et.15, Fuel Et.60+Com. Et.60, Frozen15, Frozen60, Control)
m4: y ~ treatments (Fuel Et.15+Com. Et.15, Fuel Et.60+Com. Et.60, Frozen60, Frozen15+Control)
nm: y ~ 1

N(%)

**Contrasts**	**df**	**F**	***p***	
m2 x m1	1	0.45	0.5	NS
m3 x m2	1	0.93	0.33	NS
m4 x m3	1	1.83	0.18	NS
nm x m4 (MAM)	3	224.77	<0.0001	*
Error	66			

Continues

Table S1. (Cont.)

C(%)
**Models**
m1: y ~ treatments (Fuel Et.15, Fuel Et.60, Com. Et.15, Com. Et.60, Frozen15, Frozen60, Control)
m2: y ~ treatments (Fuel Et.15+Com. Et.15, Fuel Et.60, Com. Et.60, Frozen15, Frozen60, Control)
m3: y ~ treatments (Fuel Et.15+Com. Et.15, Fuel Et.60, Com. Et.60+Control, Frozen15, Frozen60)
nm: y ~ 1

C(%)				
**Contrasts**	**df**	**F**	***p***	
m2 x m1	1	1.01	0.32	NS
m3 x m2	1	3.83	0.05	NS
nm x m3 (MAM)	3	77.1	<0.0001	*
Error				

C/N
**Models**
m1: y ~ treatments (Fuel Et.15, Fuel Et.60, Com. Et.15, Com. Et.60, Frozen15, Frozen60, Control)
m2: y ~ treatments (Fuel Et.15+Com. Et.15, Fuel Et.60, Com. Et.60, Frozen15, Frozen60, Control)
m3: y ~ treatments (Fuel Et.15+Com. Et.15, Fuel Et.60, Com. Et.60, Frozen15+Control, Frozen60)
m4: y ~ treatments (Fuel Et.15+Com. Et.15, Fuel Et.60+Com. Et.60, Frozen60, Frozen15+Control)
nm: y ~ 1

C/N				
**Contrasts**	**df**	**F**	***p***	
m2 x m1	1	0	0.99	NS
m3 x m2	1	0.01	0.96	NS
m4 x m3	1	0.32	0.57	NS
nm x m4 (MAM)	3	577.03	<0.0001	*
Error	66			
